# Diversity of *P-*element piRNA production among M' and Q strains and its association with P-M hybrid dysgenesis in *Drosophila melanogaster*

**DOI:** 10.1186/s13100-017-0096-x

**Published:** 2017-10-23

**Authors:** Keiko Tsuji Wakisaka, Kenji Ichiyanagi, Seiko Ohno, Masanobu Itoh

**Affiliations:** 1 Department of Applied Biology, Kyoto Institute of Technology, Hashigamicyo, Matsugasaki, Sakyo-ku, Kyoto, 606-8585 Japan; 2 Laboratory of Genome and Epigenome Dynamics, Department of Applied Molecular Biosciences, Graduate School of Bioagricultural Sciences, Nagoya University, Nagoya, 464-8601 Japan; 3 Center for Epidemiologic Research in Asia, Shiga Univesity of Medical Science, Otsu, Shiga 520-2192 Japan; 4 Center for Advanced Insect Research Promotion (CAIRP), Kyoto Institute of Technology, Kyoto, 606-8585 Japan

**Keywords:** Ping-pong-paired piRNA, Natural populations, Hybrid sterility, Gonadal dysgenesis, *P*-element mRNA, Progenies

## Abstract

**Background:**

Transposition of *P* elements in the genome causes P–M hybrid dysgenesis in *Drosophila melanogaster*. For the P strain, the P–M phenotypes are associated with the ability to express a class of small RNAs, called piwi-interacting small RNAs (piRNAs), that suppress the *P* elements in female gonads. However, little is known about the extent to which piRNAs are involved in the P–M hybrid dysgenesis in M′ and Q strains, which show different abilities to regulate the *P* elements from P strains.

**Results:**

To elucidate the molecular basis of the suppression of paternally inherited *P* elements, we analyzed the mRNA and piRNA levels of *P* elements in the F1 progeny between males of a P strain and nine-line females of M′ or Q strains (M′ or Q progenies). M′ progenies showed the hybrid dysgenesis phenotype, while Q progenies did not. Consistently, the levels of *P*-element mRNA in both the ovaries and F1 embryos were higher in M′ progenies than in Q progenies, indicating that the M′ progenies have a weaker ability to suppress *P*-element expression. The level of *P*-element mRNA was inversely correlated to the level of piRNAs in F1 embryos. Importantly, the M′ progenies were characterized by a lower abundance of *P*-element piRNAs in both young ovaries and F1 embryonic bodies. The Q progenies showed various levels of piRNAs in both young ovaries and F1 embryonic bodies despite all of the Q progenies suppressing *P*-element transposition in their gonad.

**Conclusions:**

Our results are consistent with an idea that the level of *P*-element piRNAs is a determinant for dividing strain types between M′ and Q and that the suppression mechanisms of transposable elements, including piRNAs, are varied between natural populations.

**Electronic supplementary material:**

The online version of this article (10.1186/s13100-017-0096-x) contains supplementary material, which is available to authorized users.

## Background

Transposable elements (TEs) occupy a substantial fraction of eukaryotic genomes, and their mobilization causes insertional mutations. Therefore, although such mobilization could provide genetic variations and drive genome evolution [1, 2], TEs could also inflict deleterious effects on the host. Piwi-interacting small RNAs (piRNAs), which are generally 23–35 nucleotides (nt) in length, suppress the expression of TEs [3]. The piRNAs can be generated via primary pathways and ping-pong biogenesis [4]. In the primary pathway, long precursor RNAs are produced from genomic loci, chopped into 23- to 35-nt RNAs (called primary piRNAs), and loaded onto the Piwi-family of protein(s). In the ping-pong biogenesis, which is known as the ping-pong amplification cycle, the piRNA-bound Piwi-family of proteins cleaves an RNA that is complementary to the bound piRNA. The cleavage occurs at the site 10-nt away from the 5′ end of the guide piRNA, and the 3′ end of the cleaved RNA is trimmed to give a 23- to 35-nt RNA (ping-pong piRNA), which are loaded onto a Piwi-family protein to guide the next round of this complementarity-based RNA cleavage. Therefore, the two RNA species (ping-pong pairs) show a characteristic 10-nt complementarity in the respective 5′ regions, referred to as a “ping-pong signature.” If a primary piRNA has a sequence antisense to a TE, it can guide the cleavage of the mRNA of the TE. Moreover, both primary and ping-pong piRNAs can guide the introduction of repressive chromatin modifications at genomic sites complementary to them. Both the primary and ping-pong biogenesis are active in germline cells in *Drosophila* [2, 5, 6] and in other organisms [4, 7]. However, in the *Drosophila* soma, only the primary pathway is utilized to generate piRNAs [8–13].

The *P* element is a DNA transposon, and their copies in the *Drosophila melanogaster* genome include structurally complete and incomplete variants. The autonomous complete elements, which are 2907 base pairs in length, encode an 87 kDa transposase that is expressed in the germline cells [14–16]. In *D. melanogaster*, crossing between females lacking *P* elements (M strain) and males carrying them (*P* strain) leads to the transposition of *P* elements in the F1 progeny (referred to as M progeny here), which causes abnormalities in the germline cells, such as gonadal dysgenesis (GD) with sterility, mutations, chromosomal breaks, and male recombination [17–20]. This phenomenon is known as P–M hybrid dysgenesis. In contrast, when *P*-strain females are mated with P-strain males, *P-*element mobilization is prevented by maternally deposited piRNAs in the germline cells and early embryos, which are laid by P-strain mothers but not P-progeny mothers (referred to as F1 embryos of P progenies) [21]. A female’s capacity to allow *P*-element transposition is defined as *P* susceptibility, which is low in the P strain but high in the M strain.

M′ and Q strains, which show different P–M phenotypes from P strains, are currently the most common in the natural populations in Eurasia, Africa, Australia, and the Far East [22–24]. Although M′ progeny allows transposition of *P* elements in the germline cells (high *P* susceptibility), the M′ strains possess many copies of *P* elements in the genome [25–27]. The Q strain carries *P* elements and have an ability to repress *P* mobilization in their progenies (low *P* susceptibility) [28–30]. In contrast to the *P* strain, males of the M′ and Q strains have no ability to induce transposition of *P* elements in their progeny (low *P* inducibility). In wild-type strains, previous studies show that *KP* elements, which are nonautonomous incomplete elements, are associated with repression [31, 32]. It has been proven that *KP* polypeptides repress *P* transposition in M′ strains [33–36]. By contrast, in both M′ and Q strains, only a weak correlation was observed between the types of genomic *P* elements and the phenotypes of the P–M system [37–40]. In our previous study, we proved that one line of M′ strain, named OM5 (see methods), have many *KP* elements in transcriptionally active sites and only a few autonomous *P* elements in inactive sites of their genomes [41]. *KP*-mediated repression and piRNA-mediated repression are also confounded [42]. Previously, it has been proved that weak piRNA-mediated repression enhances *KP*-mediated repression [43, 44]. Therefore, a major factor affecting the different *P* susceptibilities in the M′ and Q progenies remain unrevealed. It is possible that there are two hypotheses in the P–M system of M′ and Q strains as described below: (1) While neither strain contains active *P* elements to induce hybrid dysgenesis, the Q strains produce a greater number of piRNAs that enact maternal repression. (2) While M′ strains do not contain active *P* elements to induce hybrid dysgenesis, the Q strains repress dysgenesis both maternally and paternally through *KP*-mediated repression.

To study whether the production of piRNAs is involved in the difference in *P* susceptibility between M′ and Q progenies, we examined the expression levels of *P*-element piRNAs in the ovaries and whole F1 embryos. This was done by generating progenies from crossing males of a P strain and females of nine wild-type strains of the M′ or Q phenotype. We tested 2- to 3-day-old ovaries of the hybrids. These are considered to be affected by piRNAs derived from the maternally inherited *P* elements because Khurana et al. [45] showed that ovaries of 2- to 4-day-old hybrids generated by a cross between M-strain females and Har males produce no piRNAs. Moreover, the 2- to 3-day-old ovaries of hybrids were suitable for the evaluation of repression of *P* activity since they possess zygotic *P* elements from Har in their genome. Whole F1 embryos of hybrids were used for the same reasons as ovaries. The results revealed diversity in the expression levels of *P-*element piRNAs, which were correlated with mRNA expression. Importantly, we found that the production of *P*-element piRNAs was a factor dividing *P* susceptibility between the M′ and Q strains and that these piRNA production show different characters between natural strains.

## Methods

### Fly stocks

Nine isofemale *Drosophila melanogaster* lines were used: OM5, FIZ12 (FIZ-12-11), KY25 (KY-13-25), KY98 (KY-13-98), KY3 (KY-02-003), KY101 (KY-02-101), HKH (Hikone-H 1957), MSO12 (MSO-12-41), and KY74 (KY-02-074). Flies were maintained on a standard cornmeal medium at 25 °C in the laboratory throughout this investigation. The exception was for the GD test, where Harwich (Har) males and Canton S (CS) females were used as standard P and M strains, respectively. We used Har females as a control. These females had the capacity to repress paternal *P*-element transposition by maternally deposited *P*-element piRNAs [21].

### Gonadal dysgenesis (GD) test

GD tests were used to determine the strain types in the P–M system [18, 46]. Two kinds of crosses, A* (tested females × Har males) and A (CS females × tested males), were performed at 28 °C. By analyzing more than 50 F1 females for each line, the GD score was calculated as the percentage of females having dysgenic ovaries. The P–M strain type was determined based on GD scores in the cross A* (indicating susceptibility of *P* transposition) and those in the cross A (indicating *P* inducibility). The criteria for M′ strains were <10% GD in cross A and >10% GD in cross A*. The criteria for Q strains were <10% GD in both crosses [47] (see Table 1). KY25, KY98, MSO12, and FIZ12 were tested first. We retested KY3, HKH, KY101, KY74, and OM5, because these lines had undergone many generations since the previous GD tests [48].

**Table 1:** Strain types in the P-M system

*P* susceptibility high: >10%GD low: <10%GD	*P* inducibility low: <10%GD high: >10%GD	strain type
high	low	M’
low	low	Q
low	high	P
high	low	M (*P-*elements (−))

*Drosophila melanogaster* is divided into the four strain types by GD ratios. *P* susceptibility shows the regulatory capacity against the *P-*elements and *P* inducibility exhibits the ability to transpose *P-*elements in progeny

### RNA preparation

To accurately analyze the correlation between the number of *P*-element piRNAs and the expression level of *P*-element mRNA, both small RNAs and total RNAs were prepared from same sample, as described below. Total RNA was extracted from 2- to 3-day-old ovaries or 0- to 24-h F1 embryos with the miRNeasy kit (Qiagen). Small RNAs were separated using the RNeasy MinElute Cleanup Kit (Qiagen). 0- to 24-h embryos were generated by 30–40 couples of cross A* kept in bottles on dishes. Eight ovaries of 2- to 3-day-old F1 females were dissected. These ovaries were generated by approximately 20 couples kept in bottles for 4–7 days at the GD-inducing temperature of 28 °C [18, 46 were arranged]. In OM5 × Har, we used equal numbers of complete and dysgenic ovaries.

### Small RNA sequencing

The small RNA libraries were produced using 1 μg of small RNAs with the Truseq small RNA sample preparation kit (Illumina). After PCR amplification, products of approximately 150 bp were collected from a 6% polyacrylamide gel. Single-end 50-bp sequencing of these libraries was carried out on MiSeq (Illumina).

Analysis of the obtained piRNA sequence was performed as previously described [21, 8, 45] using the CLC Genomics Workbench (detailed protocol is described in https://www.qiagenbioinformatics.com/support/manuals/). After trimming of the adaptor sequence by Transcriptomics Analysis in g_x_, we removed the reads corresponding to 2SrRNA, which were included in a considerable ratios (average of 92% of total reads). To see how much of the sequencing libraries corresponded to 2SrRNAs, we examined the number of total reads, 23- to 30-nt piRNAs and 186 TE-derived 23- to 30-nt piRNAs (Additional file 1: Table S1). Reads that were mapped to rRNAs, tRNAs, and snoRNAs were removed. The remaining reads were mapped to the *D. melanogaster* genome (Release R22) using Download Genome in g_x_. RNA reads of 23–35 nts that did not match miRNA sequences in miRBase [49] were defined as piRNAs. These sequences were then mapped to *P-*element sequences [14] and 186 transposons (total TEs) (Repbase) by Map Reads to Reference in g_x_. For normalization across the samples, the read numbers of piRNAs mapped to *P* elements were divided by the total number of miRNA reads and multiplied by 1 million. This gave the reads per million (RPM miRNA reads). Ping-pong signatures were analyzed by per scripts [3, 50, 51].

### RT-PCR and quantitative RT-PCR

cDNA was synthesized by superscript III reverse transcriptase (Invitrogen) using total RNA and oligo-dT primer. Quantitative amplification of cDNA was performed in duplicate using SYBR Green quantitation (Toyobo) on a 7000 HT Fast Real-Time PCR System (Applied Biosystems; forward and reverse primers: 5′-GTGGGAGTACACAAACAGAGTCCTG-3′ and 5′-CGTATCTGCGTGTCCGTGAAGA-3′). The level of *P*-element mRNA was normalized to that of RP49 mRNA (forward and reverse primers: 5′-CGGATCGATATGCTAAGCTGT and 5′-GCGCTTGTTCGATCCGTA) [52].

### Statistical analysis

**Fig. 1:**
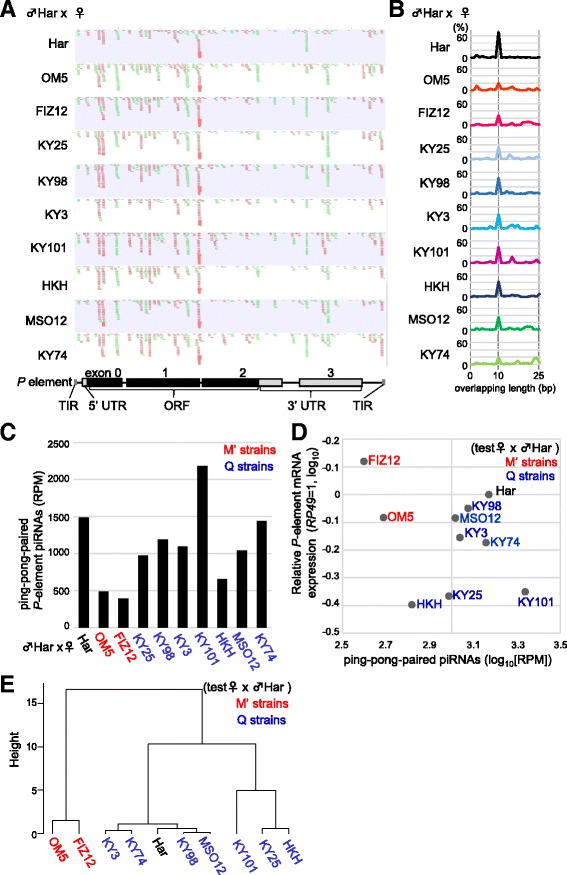
Expression of piRNA and mRNA of *P* elements in adult ovaries of F1 progenies in cross A*. **a** Small RNA reads (23–35 nt in length) mapped to the sense (green) and antisense (red) strands of the *P* element are shown on the *P*-element structure (bottom). Har (top) was a P strain and used as a control. **b** Frequencies of length (0–25 bp, *x*-axis) of overlapping regions between sense and antisense small RNAs (23–35 nt) identified in ovaries of F1 progenies. An overlap of 10 bp is a signature of piRNA pairs produced via the ping-pong cycle. **c** The expression levels of ping-pong-paired piRNAs in F1 ovaries normalized by miRNA (reads per million [RPM] miRNAmiRNA reads). The strain names of mothers are shown in black (P), red (M′), and blue (Q). **d** The relationship between the log expression levels of mRNAs (*y*-axis) and ping-pong-paired piRNAs (*x*-axis) of *P* elements in F1 ovaries. The strain names of mothers are shown in black (P), red (M′), and blue (Q). The Pearson’s correlation efficient is shown on the top. **e** A tree of hierarchical clustering of the nine natural strains and the Har strain based on the data shown in panel **c**. The strain names of mothers are shown in black (P), red (M′), and blue (Q). The M′ strains are clustered together

The Pearson product-moment correlation test and hierarchical cluster analysis were performed using R. For the hierarchical cluster analyses in Figs. 1e and 3b, we used the hclust function in R (ver. 3.0.2) with the furthest neighbor method.

## Results

### GD test revealed two lines of M′ and seven lines of Q strains

To test their capacity to regulate the paternally inherited *P* elements in F1 ovaries, females of nine natural strains were crossed to Har males (P strain) having high *P* inducibility (cross A*). The GD scores (fraction of their daughters showing dysgenic ovaries, see Methods) in cross A* indicate the *P* susceptibility of the test strain (Table 1). F1 progeny of KY25, KY98, KY3, KY101, HKH, MSO12, and KY74 displayed GD scores of 0 to 10%, indicating that *P*-element transposition was highly repressed in their ovaries (Table 2). In contrast, OM5 and FIZ12 showed GD scores of more than 10%, indicating *P*-element transposition activity in their ovaries. We also analyzed ovaries of F1 progeny from cross A, where males of each strain were crossed to CS females (M stain) with *P* susceptibility. In all tests, F1 progeny displayed GD scores less than 1% (Table 2), indicating that *P* inducibility is very limited in the nine strains.

**Table 2:** GD ratios and total *P*-element piRNAs production in the progeny

Test strain	GD^a^ (%)	GD^a^ (%)	Deduced strain type	*P*-element piRNAs (RPM)^bb^		total-TE piRNAs (RPM)^bb^	
	cross A*	cross A		cross A*		cross A*	
	(♀test x ♂Har)	(♀CS x ♂test)		(♀test x ♂Har)		(♀test x ♂Har)	
				Ovaries	F1 embryos	Ovaries	F1 embryos
OM5	28.3	0	M’	5137	233	1,105,901	139,280
FIZ12	13.3	1	M’	6108	522	1,158,677	138,991
KY25	0	0	Q	7333	740	1,428,653	130,670
KY98	0	1	Q	9356	830	1,502,704	143,827
KY3	2.5	0	Q	8049	818	1,060,199	144,297
KY101	0	0	Q	18,009	1662	1,958,902	109,059
HKH	0	0	Q	4989	2421	1,048,057	417,233
MSO12	0	0	Q	8941	5200	1,351,448	521,266
KY74	0.8	0	Q	9077	6336	1,343,427	425,912
Har	0	100	P	7780	604	1,090,850	99,772

^a^Percentage of gysgenic ovaries from cross A* (test female x Har male) and cross A (CS female x test male). ^b^piRNA reads were divided by miRNA reads, expressed as reads per million miRNA reads (RPM) in the progeny from cross A*

We classified these nine lines into two types according to the GD scores. Seven strains (KY25, KY98, KY3, KY101, HKH, MSO12, and KY74) showed low *P* susceptibility and low *P* inducibility, and thus they were Q strains. The other two strains (OM5 and FIZ12) were classified as M′ strains due to their high *P* susceptibility and low *P* inducibility.

### Various levels of ping-pong-paired piRNAs derived from *P* elements in ovaries of young dysgenic progenies

The GD test above showed that progenies from the M′ strains (M′ progenies) displayed higher *P* susceptibilities than those from the Q strain (Q progenies) and the P strain (P progenies). To examine the possibility that this variation is due to the difference in the expression level of *P*-element piRNAs in germline cells of the F1 progenies, we performed deep sequencing of small RNAs present in the ovaries of 2- to 3-day-old progenies of crosses between Har males and M′ or Q females. After removal of miRNAs and fragments of functional RNAs, small RNAs of 23- to 35 nt in length were mapped to the sequences of *P* elements to identify *P*-element piRNAs (Fig. 1a).

In all cases, we detected *P*-element-derived piRNAs in both sense and antisense directions. These piRNAs were mapped mainly to exons 0 and 1, showing that there is some sequence similarity between lines. The M′ progenies (OM5 and FIZ15) produced the lowest numbers of piRNAs compared with the Q and P progenies, except for HKH. Such a low abundance was specific to the *P* element because the total TE-derived piRNAs in the M′ progenies were comparable with those in others (Table 2). To study whether the detected piRNAs are generated via ping-pong biogenesis in germline cells, we analyzed the overlap between sense and antisense piRNAs (Fig. 1b). Indeed, a peak at 10 bp was evident in all cases, which suggested that a substantial fraction of the piRNAs were produced via ping-pong biogenesis. Interestingly, abundance of ping-pong-paired piRNAs were less in the M′ progenies compared with the Q and P progenies, suggesting that the ability of M′ progenies to amplify and maintain piRNAs in the germline cells is weaker than that of Q and P progenies (Fig. 1c). The Q progenies expressed various amounts of ping-pong piRNAs. These amounts were comparable with those in the P progenies and highlight that the higher ability to repress the *P* element is associated with a higher expression of ping-pong-paired piRNAs in the ovaries. In particular, KY101 progenies showed quite high amounts of ping-pong-paired piRNAs produced from *P* elements.

We next determined the levels of *P*-element mRNA in these ovaries by reverse transcription followed by quantitative PCR (qRT-PCR). The average expression levels of ovarian *P*-element mRNA was 0.1-fold lower than in embryonic *P*-element mRNA in 10 progenies. The mRNA levels varied between the progenies, with a tendency for the M′ progenies to show higher expression than the Q progenies (Fig. 1d). Furthermore, we repeated the qRT-PCR three to five times in four lines of M′ and Q strains and ensured that there was significantly higher expression of *P*-element mRNA in M′ (OM5) progenies compared with that in Q progenies (KY3, KY101 and KY74; *p* = 0.03, 0.003 and 0.05, respectively; Additional file 2: Figure S1). However, ovaries of KY3 (Q) progenies showed a high score of standard division (SD = 0.3). This suggests that individuals of KY3 progenies differ in their expression level of *P* elements. Importantly, the two M′ progenies were clustered in hierarchical clustering of *P*-element mRNA and *P*-element ping-pong piRNA expression levels (Fig. 1e). These results favor an idea that the level of ping-pong-paired piRNAs is one determining factor for the expression level of *P* elements in natural populations.

M′ progenies were characterized by a low ability to produce ping-pong-paired piRNAs and high levels of *P*-element expression in the ovaries. While Q progenies were distinguished from M′ progenies by the amount of ping-pong-paired piRNAs and the levels of *P*-element expression, they showed variable levels of expression of piRNAs and mRNA.

### Various levels of ping-pong-paired piRNAs derived from *P* elements in F1 embryos of progenies

**Fig. 2:**
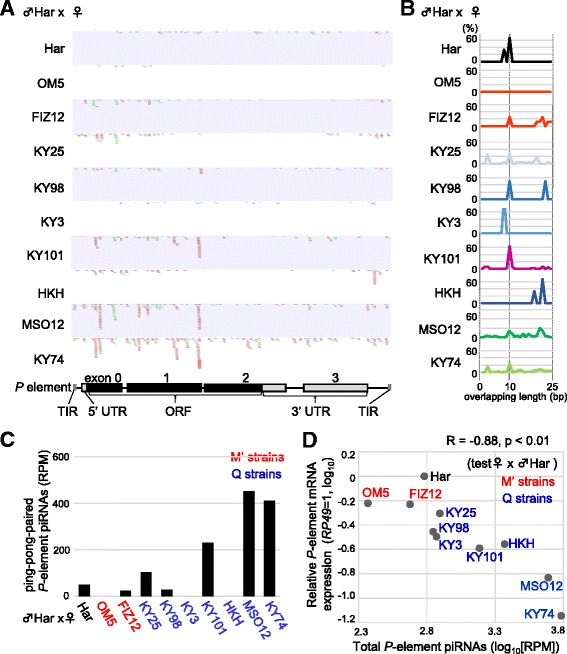
Expression of piRNA and mRNA of *P* elements in F1 embryonic bodies of F1 progenies in cross A*. **a** Small RNA reads (23–35 nt) mapped to the sense (green) and antisense (red) strands of the *P* element are shown on the *P*-element structure (bottom). Har (top) was a P strain as a control. **b** Frequencies of length (0–25 bp, *x*-axis) of overlapping regions between sense and antisense small RNAs (23–35 nt) identified in F1 embryos. An overlap of 10 bp is a signature of piRNA pairs produced via the ping-pong cycle. **c** The expression levels of ping-pong-paired piRNAs in F1 ovaries (reads per million [RPM] miRNa reads). The strain names of mothers are shown in black (P), red (M′), and blue (Q). **d** The relationship between the log expression levels of mRNAs (*y*-axis) and piRNAs (*x*-axis) of *P* elements in F1 ovaries. The strain names of mothers are shown in black (P), red (M′), and blue (Q). The Pearson’s correlation efficient is shown on the top

To study the possible involvement of piRNAs in the regulation of the paternally inherited *P* elements during embryogenesis of the F1 progeny, we next analyzed *P*-element piRNAs and mRNA in whole F1 embryos (<24 h after hatching) of progenies of cross A*. It has been proven that *P*-element piRNAs produced in F1 embryos of hybrids between M-strain females and Har are very limited [45]. In contrast, we detected *P*-element piRNAs in whole F1 embryos of M′, Q, and P progenies (Table 2). There was a considerable variation in the abundance. The M′ progenies again showed the lowest abundance of *P*-element piRNAs although they produced total TE-derived piRNAs at levels similar to those in the Q and P progenies (Table 2). Analysis of sense and antisense piRNAs revealed that ping-pong-paired piRNAs are generally lower in whole F1 embryonic bodies than in ovaries. In particular, the two M′ progenies, in addition to KY98, KY3, and HKH progenies, produced a fewer number of ping-pong-paired piRNAs (Fig. 2b and c). It is possible that some of the strange discrepancies with ovarian piRNAs from the same lines are caused by the limited power to accurately estimate the ping-pong fraction. This could be due to the production level of total-TE-derived piRNAs in F1 embryonic bodies being less than those in the ovaries (Table 2). Therefore, the level of total *P*-element piRNAs was evaluated to compare differences between lines, as below.

We investigated whether the expression of *P*-element mRNA was associated with the production of piRNAs derived from *P* elements in whole F1 embryos of the natural strains. We quantified *P*-element mRNA in the F1 embryonic bodies. This revealed that *P*-element expression is somewhat higher (not significantly) in M′ progenies compared with Q progenies (Fig. 2d). We repeated qRT-PCR three times in five lines of M′, Q, and P strains and ensured that there was a significantly higher expression of *P*-element mRNA in M′ (OM5) progenies compared with those in the Q progenies (KY3, KY101, and KY74; *p* < 0.05) (Additional file 3: Figure S2A and B). Furthermore, 10 lines were classified into P, M′, and Q strains, and it was determined that the mRNA expression level was negatively correlated to the expression level of total *P*-element piRNAs (*R* = −0.88, *p* < 0.01; Fig. 2d). We made sure that this negative correlation between the total *P*-element piRNAs and the mRNA level was analyzed by three biological replicates for five progenies (*R* = −0.9, *p* < 0.05; Additional file 3: Figure S2). These results suggest that cells in the F1 embryonic bodies produce piRNAs mainly via the primary pathway and that these primary piRNAs play a role in *P*-element regulation during embryogenesis.

### M′ strains were characterized by the lowest production of ping-pong-paired piRNAs in both young adult ovary and F1 embryonic bodies

**Fig. 3:**
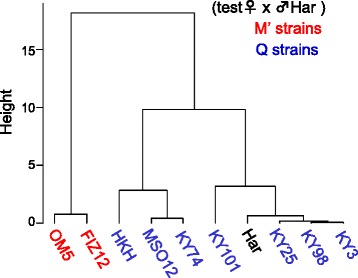
Characterization of the natural strains based on piRNA levels in F1 progenies. Relationship between the expression levels (RPM) of *P*-element ping-pong-paired piRNAs in F1 ovaries and total *P*-element piRNAs in embryos. Hierarchical clustering of the nine strains and the Har

The above results showed a tendency that ping-pong-paired *P*-element piRNAs in the ovary and the total *P*-element piRNAs in F1 embryos are less in the M′ progenies than in the Q and P progenies. To reveal whether there were clear differences in the amount of piRNAs derived from *P* elements between the M′ progenies and others, we did clustering analysis *P*-element ping-pong piRNAs production in the ovaries and total *P*-element piRNAs in F1 embryos of progenies. Actually, M′ progenies were characterized by the lowest production of *P*-element piRNAs in both the young adult ovary and in F1 embryonic bodies. For the Q and P progenies, KY101, Har, KY25, KY98, and KY3 showed higher production of *P*-element *P*-element piRNAs in young adult ovaries, while HKH, MSO12, and KY74 produced higher levels of *P*-element piRNAs in the F1 embryos (Fig. 3).

## Discussion

Although the natural population of *D. melanogaster* generally carries *P* elements in their genome, the progeny displays a different resistance capacity against *P* elements as introduced upon hybridization with typical P strains. Here, we showed that the M′ strains distinguished from the Q strains by low levels of *P*-element piRNA production in both the ovaries and the F1 embryos of dysgenic progenies, and that this is associated with a low ability to suppress *P*-element transcription. This character of M′ strains is likely related to their high level of GD, which is linked to *P*-element transposition. In contrast, it was shown that the Q progenies produced various degrees of *P*-element piRNAs. This could confer the ability to resist *P*-element expression in embryonic bodies. However, such varied production of *P*-element piRNAs among Q progenies did not induce different levels of GD.

Interestingly, M′ progenies of the two lines, which showed moderate scores of GD in cross A*(10%–30%) indicating partial repression of *P* transposition, produced *P*-element piRNAs in young adult ovaries at some degree. In I–R hybrid dysgenesis, the levels of *I-*element piRNAs inversely correlated with dysgenic scores [53]. While it has been reported that other repressive factors for *P*-element transposition, such as proteins produced from full-length (type I, 66-kDa repressors) and internally deleted elements (type II, *KP* repressors), play a role in germline cells to some degree, our results suggest that the level of *P*-element piRNAs in the M′ progenies is one major determinant of the *P* susceptibility, which is in addition to the P–M phenotype in M′ strains, as shown in the I–R system. Further studies are necessary to investigate M′ strains having various levels of *P* susceptibility. Why the M′ progenies are not able to produce abundant *P*-element piRNAs despite the presence of *P* elements in their maternal genomes? It is thought that piRNAs are inherited from the oocytes of the mothers and is imparted to the F1 progenies. These inherited piRNAs act to prime the ping-pong amplification cycle in the germline cells of the daughters. Thus, it is possible that the maternal lineage of the M′ strains does not produce abundant piRNAs. To produce both primary and ping-pong piRNAs, a genomic situation is required where *P* element(s) are located in the piRNA clusters [20]. Therefore, the copy number of *P* elements in the piRNA clusters is likely less in the genomes of the M′ strains, resulting in a reduced level of *P*-element piRNA production. Previously, it has been proven that autonomous complete *P* elements in M′ strains are transcriptionally inactive [41]. Therefore, the other possibility is that such *P* elements are repressed in M′-strain parents and may not contribute to resistance against *P* elements introduced upon hybridization with typical P strains. Future studies, such as piRNA profiling of oocytes of mothers, will evaluate these possibilities.

For the Q strains, despite their resistance to paternal *P* elements, there was considerable variation in the mRNA and piRNA expression levels of *P* elements in both the ovary and the F1 embryonic bodies. Therefore, in Q strains, the molecular basis of production of *P*-element piRNAs affecting the P–M phenotype is likely different from that in I–R hybrid dysgenesis. In particular, progeny of KY101 showed higher production of *P*-element ping-pong-paired piRNAs in the ovaries, suggesting that piRNAs act as a main suppressor during oogenesis. F1 embryos of MSO12 and KY74 progenies produced abundant *P*-element piRNAs, including ping-pong-paired piRNAs, and lower levels of *P*-element mRNA. This suggested that piRNAs act as one of the main suppressors during embryogenesis. Other Q progenies were classified into two groups that were characterized by KY101 and KY74, as discussed above. They allowed the expression of the *P*-element mRNA at levels similar to those in the M′ progenies. This would imply that other factors, such as protein repressors, are involved in the repression of *P*-element transposition in the Q progenies [54]. It is also possible that individuals could differ in their sensitivity to germline P activity (higher for M′ progenies and lower for Q progenies), resulting in different severities of hybrid dysgenesis under equivalent levels of transpositional activity. Furthermore, whole F1 embryos are composed of germ line cells producing ping-pong piRNAs and somatic cells producing antisense piRNAs. Thus, further studies are required to address the varied expression of both *P*-element piRNAs and mRNA in Q progenies, including the effect from embryonic somatic cells and germ line cells. Interestingly, Har progeny was in the same group as KY101 progeny, which showed a higher production of *P*-element ping-pong-paired piRNAs in the ovaries. It is possible that those Q and P progenies have *P* elements inserted into germ-specific piRNA clusters, which produce ping-pong-paired piRNAs. Thus, in the ovaries of Q and P progenies, ping-pong-paired piRNAs likely act to suppress *P* elements introduced upon hybridization with typical *P* strains. On the other hand, males of the P strain have a high ability to mobilize *P* elements in their progeny when they are mated with M-strain females; this is in contrast to what is found in the Q strain. Therefore, the P strain may possess many *P* elements in active expression sites of the genome. Another possibility is that the P strain produces lower levels of zygotic piRNAs derived from paternal *P* elements. More investigation into the insertion site of *P* elements and *P* inducibility is required. Furthermore, since *P*-element-derived piRNAs exhibited similar sequences in all lines, piRNA biogenesis may not differ between lines.

## Conclusions

Our results suggest that piRNA abundance explains coarse phenotypic differences between M’ and Q cytotypes with respect to P-repression, but not more modest differences between Q strains. Whether this piRNA variation originates from genetic diversity, such as copy number and location of *P* elements, or from long-term inheritance of small RNAs may be an interesting question. Moreover, our results evoke an interesting possibility that the suppression mechanisms of TEs including piRNAs are varied in natural populations.

## Additional files

Additional file 1: Supplemental methods. Table S1. (DOCX 18 kb)

Additional file 2: Figure S1. Expression of mRNA of *P* elements in F1 ovaries of progenies of four lines. (PPTX 45 kb)

Additional file 3: Figure S2. Expression of piRNA and mRNA of *P* elements in F1 embryonic bodies of progenies of file lines. (PPTX 52 kb)

## Abbreviations

cross A: CS females x tested males
cross A*: Tested females x Har males
CS: Canton S
GD: Gonadal dysgenesis
Har: Harwich
low *P* inducibility: The ability to induce transposition of *P* elements in the germline cells of progeny
M, P, M′, or Q progeny: The F1 progeny between males of a P strain and females of each strain
*P* susceptibility: The capacity to allow transposition of *P* elements in the germline cells of progeny
piRNAs: Piwi-interacting small RNAs
qRT-PCR: Quantitative PCR
RPM: Reads per million miRNA reads
TEs: Transposable elements
