# Supplementary material for: Diversity of P-element piRNA production among M' and Q strains and its association with P-M hybrid dysgenesis in Drosophila melanogaster
Source: Mob DNA. 2017 Oct 23;8:13. doi: 10.1186/s13100-017-0096-x (PMC5654125; doi:10.1186/s13100-017-0096-x)
Supplement: Supplementary file 1 — Supplemental methods. Table S1. (DOCX 18 kb) [file 13100_2017_96_MOESM1_ESM.docx]

**Additional file 1**

**Supplemental methods**

**RNA preparation**

To confirm the expression levels of *P*-element mRNA, we analyzed the mRNA level of three to five biological replicates for four to five lines of M′, Q, and P strains. Total RNA was extracted from 2- to 3-day-old ovaries or 0- to 24-h embryos with the NucleoSpin RNA Plus (MACHEREY-NAGEL). 0- to 24-h embryos were generated by 10–30 couples of cross A* kept in bottles on dishes. Five to eight ovaries of 2- to 3-day-old F1 females were dissected. These ovaries were generated by approximately 20 couples kept in bottles for 4–7 days at the GD-inducing temperature of 28°C (Engels and Preston 1979; Kidwell and Novy 1979 were arranged).

**Statistical analysis**

The Pearson product-moment correlation test and Student’s t-test were performed.

**Table S1**

**The read numbers of total sequence, and the read numbers and read fractions of 2S rRNA, 23-30 nt piRNAs, and 186 TE-derived piRNAs**

|  | read number | | | | | |  | read % | | | |
| --- | --- | --- | --- | --- | --- | --- | --- | --- | --- | --- | --- |
|  | total | 2s rRNA | piRNAs (23-30 nt) | | | TE-derived piRNAs (23-30 nt) |  | 2s rRNA | piRNAs (23-30 nt) | | TE-derived piRNAs (23-30 nt) |
|  |  |  |  |  |  |  |  |  |  |  |  |
|  |  |  |  |  |  |  |  |  |  |  |  |
| ovaries |  |  | |  |  | |  |  |  |  | |
| ♀ x H♂ | |  | |  |  | |  |  |  |  | |
| Har | 2394769 | 2253451 | | 67404 | 39253 | |  | 94 | 2.8 | 1.7 | |
| OM | 6644707 | 6271258 | | 178880 | 109483 | |  | 94 | 2.7 | 1.7 | |
| FIZ 12 | 5537888 | 5248286 | | 140225 | 85291 | |  | 95 | 2.5 | 1.6 | |
| KY 25 | 3922287 | 3648928 | | 141968 | 85657 | |  | 93 | 3.6 | 2.3 | |
| KY 98 | 3316795 | 3064388 | | 133035 | 81845 | |  | 92 | 4 | 2.7 | |
| KY 3 | 2635974 | 2521614 | | 55692 | 33201 | |  | 96 | 2.1 | 1.3 | |
| KY 101 | 3589270 | 3327561 | | 155378 | 101460 | |  | 93 | 4.3 | 3 | |
| HKH | 6563901 | 6309277 | | 123091 | 70909 | |  | 96 | 1.9 | 1.1 | |
| MSO 12 | 2818614 | 2629484 | | 101792 | 60633 | |  | 93 | 3.6 | 2.3 | |
| KY74 | 4488113 | 4167013 | | 153630 | 93321 | |  | 93 | 3.4 | 2.2 | |
| embryos |  |  | |  |  | |  |  |  |  | |
| ♀ x H♂ | |  | |  |  | |  |  |  |  | |
| Har | 3698693 | 3303856 | | 45842 | 4672 | |  | 89 | 1.2 | 0.1 | |
| OM | 3601284 | 3322555 | | 30867 | 4054 | |  | 92 | 0.9 | 0.1 | |
| FIZ 12 | 4297853 | 3889880 | | 83370 | 16930 | |  | 91 | 1.9 | 0.4 | |
| KY 25 | 3974450 | 3553864 | | 82698 | 13314 | |  | 89 | 2.1 | 0.4 | |
| KY 98 | 3296525 | 2997291 | | 61461 | 9404 | |  | 91 | 1.9 | 0.3 | |
| KY 3 | 2567022 | 2339107 | | 26513 | 3271 | |  | 91 | 1 | 0.1 | |
| KY 101 | 2838543 | 2531023 | | 57529 | 8101 | |  | 89 | 2 | 0.3 | |
| HKH | 2381648 | 2136001 | | 116498 | 8293 | |  | 90 | 4.9 | 0.4 | |
| MSO 12 | 3933562 | 3613258 | | 80847 | 20358 | |  | 92 | 2.1 | 0.6 | |
| KY74 | 4607287 | 4163118 | | 98490 | 24251 | |  | 90 | 2.1 | 0.6 | |
