# Supplementary material for: Diversity of P-element piRNA production among M' and Q strains and its association with P-M hybrid dysgenesis in Drosophila melanogaster
Source: Mob DNA. 2017 Oct 23;8:13. doi: 10.1186/s13100-017-0096-x (PMC5654125; doi:10.1186/s13100-017-0096-x)
Supplement: Supplementary file 2 — Expression of mRNA of P elements in F1 ovaries of progenies of four lines. (PPTX 45 kb) [file 13100_2017_96_MOESM2_ESM.pptx]

## Slide 1
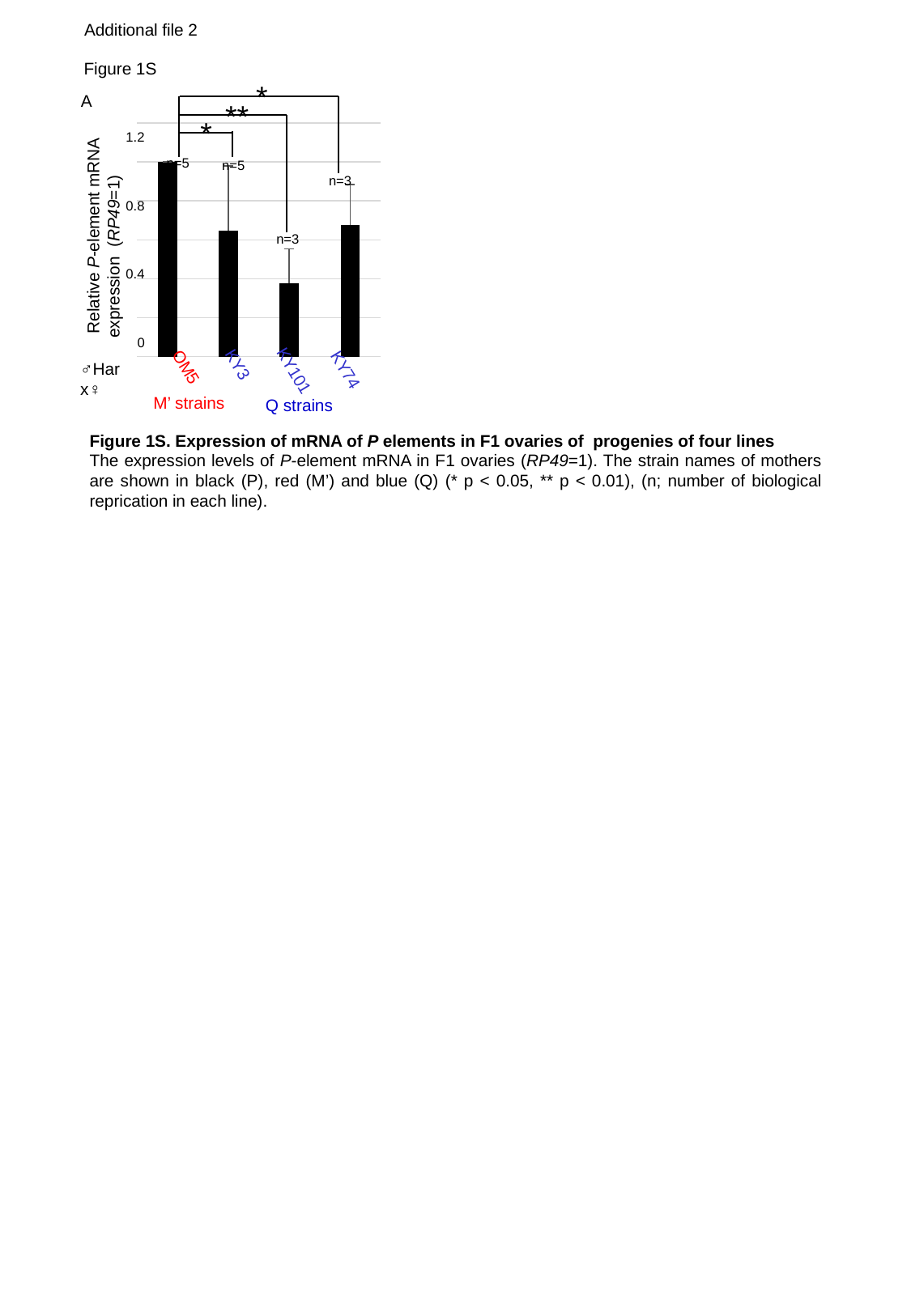

Additional file 2
Figure 1S
*
A
**
*
### Chart
| Category | |
|---|---|
| omh | 1.0 |
| 3h | 0.6465499855927018 |
| 101h | 0.37851121528710846 |
| 74h | 0.6760789467300087 |1.2
n=5
n=5
n=3
0.8
 Relative P-element mRNA expression (RP49=1)
n=3
0.4
0
KY3
OM5
♂Har x♀
KY74
KY101
M’ strains
Q strains
Figure 1S. Expression of mRNA of P elements in F1 ovaries of progenies of four lines
The expression levels of P-element mRNA in F1 ovaries (RP49=1). The strain names of mothers are shown in black (P), red (M’) and blue (Q) (* p < 0.05, ** p < 0.01), (n; number of biological reprication in each line).
