# Supplementary material for: Diversity of P-element piRNA production among M' and Q strains and its association with P-M hybrid dysgenesis in Drosophila melanogaster
Source: Mob DNA. 2017 Oct 23;8:13. doi: 10.1186/s13100-017-0096-x (PMC5654125; doi:10.1186/s13100-017-0096-x)
Supplement: Supplementary file 3 — Expression of piRNA and mRNA of P elements in F1 embryonic bodies of progenies of file lines. (PPTX 52 kb) [file 13100_2017_96_MOESM3_ESM.pptx]

## Slide 1
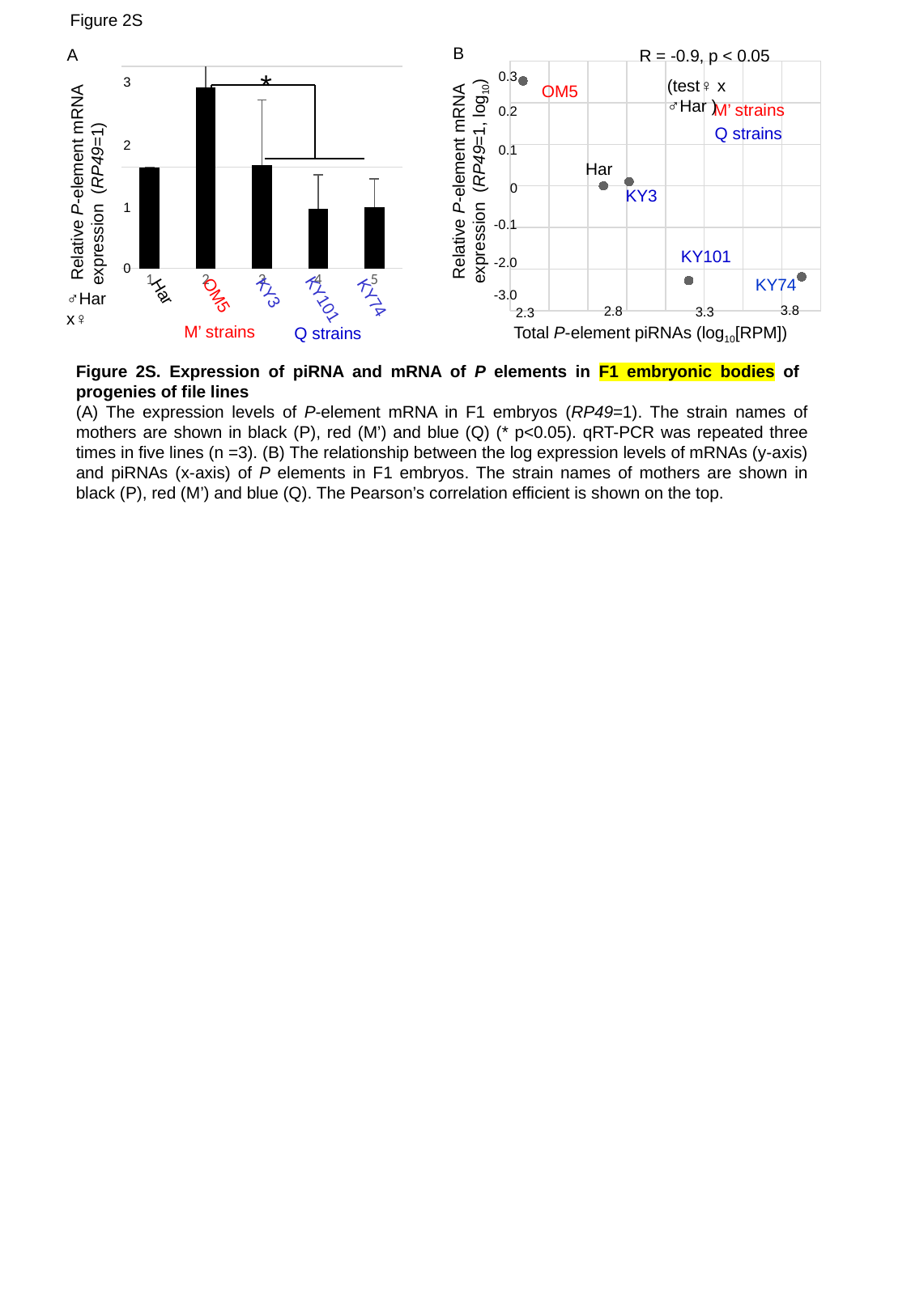

Figure 2S
B
A
R = -0.9, p < 0.05
### Chart
| Category | |
|---|---|*
### Chart
| Category | |
|---|---|0.3
3
(test♀ x ♂Har )
OM5
M’ strains
0.2
Q strains
2
0.1
 Relative P-element mRNA expression (RP49=1, log10)
Har
 Relative P-element mRNA expression (RP49=1)
0
KY3
1
-0.1
KY101
-2.0
0
KY74
Har
KY3
OM5
-3.0
♂Har x♀
KY74
KY101
3.8
2.8
3.3
2.3
M’ strains
Total P-element piRNAs (log10[RPM])
Q strains
Figure 2S. Expression of piRNA and mRNA of P elements in F1 embryonic bodies of progenies of file lines
(A) The expression levels of P-element mRNA in F1 embryos (RP49=1). The strain names of mothers are shown in black (P), red (M’) and blue (Q) (* p<0.05). qRT-PCR was repeated three times in five lines (n =3). (B) The relationship between the log expression levels of mRNAs (y-axis) and piRNAs (x-axis) of P elements in F1 embryos. The strain names of mothers are shown in black (P), red (M’) and blue (Q). The Pearson’s correlation efficient is shown on the top.
